# Supplementary material for: Label-Free SERS and MD Analysis of Biomarkers for Rapid Point-of-Care Sensors Detecting Head and Neck Cancer and Infections
Source: Sensors (Basel). 2023 Nov 2;23(21):8915. doi: 10.3390/s23218915 (PMC10648186; doi:10.3390/s23218915)
Supplement: Supplementary file 1 [file sensors-23-08915-s001.zip › sensors-2616354-supplementary.pdf]

Supporting Information

Label-free SERS and MD analysis of salivary biomarkers for rapid POC sensors detecting head neck cancer and infections

Edoardo Farnesi[1,2], Silvia Rinaldi[3], Chen Liu[1,2], Jonas Ballmaier[4], Orlando Guntinas-Lichius[4], Michael Schmitt[1], Dana Cialla-May[1,2] and Jürgen Popp[1,2]

[1] Institute of Physical Chemistry (IPC) and Abbe Center of Photonics (ACP), Friedrich Schiller University Jena, Member of the Leibniz Centre for Photonics in Infection Research (LPI), Helmholtzweg 4, 07743 Jena, Germany

[2] Leibniz Institute of Photonic Technology, Member of Leibniz Health Technologies, Member of the Leibniz Centre for Photonics in Infection Research (LPI), Albert-Einstein-Straße 9, 07745 Jena, Germany

[3] Institute for the Chemistry of Organo Metallic Compounds, National Research Council of Italy (CNR), Via Madonna del Piano 10, Sesto Fiorentino, 50019 Florence, Italy

[4] Department of Otorhinolaryngology-Head and Neck Surgery, Jena University Hospital, 07747 Jena, Germany

Table S1. Detailed informations about the individuals providing the saliva sample.

ID	GENDER	YOB	STATUS
#1	Male	1972	Healthy
#2	Male	1969	Healthy
#13	Female	1962	Healthy

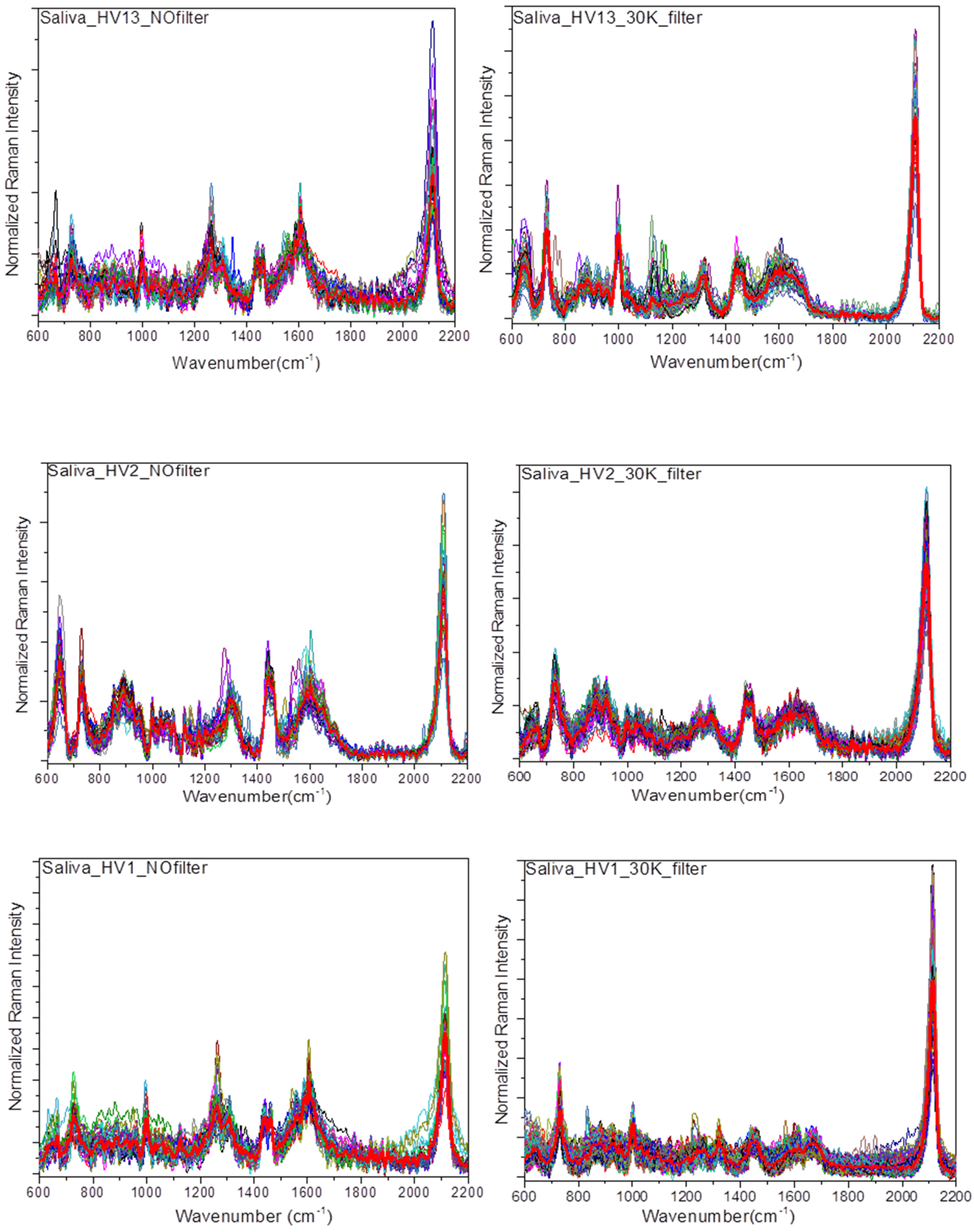

Figure S1. Comparison between no-filter (left column) and 30kDa cut-off filter (right column) background-corrected SERS spectra of saliva from 3 healthy volunteers. For each sample 250 spectra were acquired, elaborated (as reported in Methods section) and the average spectrum (red line) was plotted.

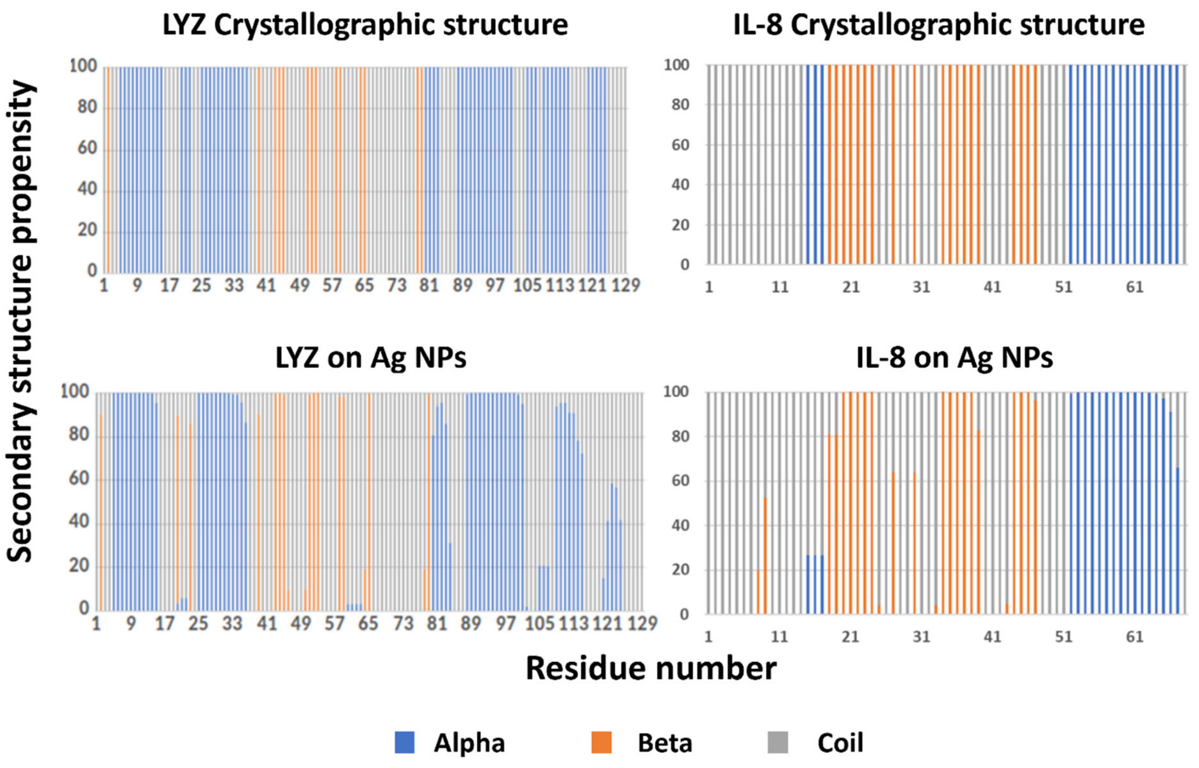

Figure S2. Secondary structure propensity computed along the simulation time and in the reference crystallographic structure for the Lys and IL-8 system.

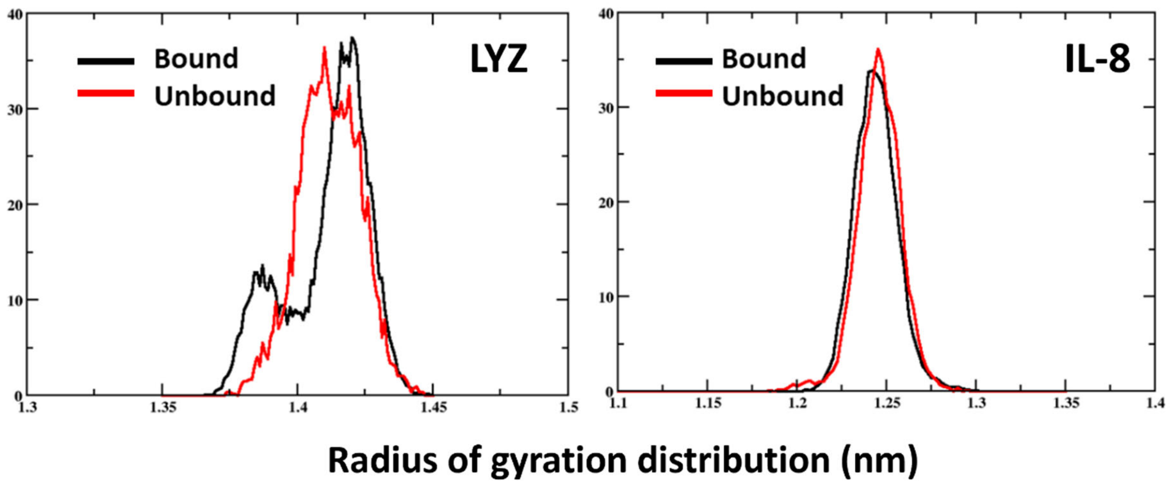

Figure S3. Radius of gyration distribution computed on Lys and IL-8 system in the presence and in the absence of silver nanoparticles.
